# Supplementary material for: A 3D adult zebrafish brain atlas (AZBA) for the digital age
Source: eLife. 2021 Nov 22;10:e69988. doi: 10.7554/eLife.69988 (PMC8639146; doi:10.7554/eLife.69988)
Supplement: Supplementary file 1. [file elife-69988-supp1.docx]

Supplementary File 1: Brain region abbreviations, full names, and colors

| **Abbreviation** | **Full name** | **Color** |
| --- | --- | --- |
| A | anterior thalamic nucleus |  |
| AC | anterior cerebellar tract |  |
| ALLN | anterior lateral line nerves |  |
| AON | anterior octaval nucleus |  |
| APN | accessory pretectal nucleus |  |
| ATN | anterior tuberal nucleus |  |
| BSTa | bed nucleus of the stria terminalis, anterior division |  |
| BSTm | bed nucleus of the stria terminalis, medial division |  |
| BSTpd | bed nucleus of the stria terminalis, posterior division |  |
| C | central canal |  |
| Cans | ansulate commissure |  |
| Cantd | anterior commissure, dorsal part |  |
| Cantv | anterior commissure, ventral part |  |
| CC | cerebellar crest |  |
| CCe-g | cerebellar corpus, granular layer |  |
| CCe-m | cerebellar corpus, molecular layer |  |
| Ccer | cerebellar commissure |  |
| Cgus | commissure of the secondary gustatory nuclei |  |
| Chab | habenular commissure |  |
| Chor | horizontal commissure |  |
| CIL | central nucleus of the inferior lobe |  |
| Cinf | commissura infima of Haller |  |
| CM | mammillary body |  |
| CO/OT | optic chiasm / optic tract |  |
| CON | caudal octavolateralis nucleus |  |
| CP | central posterior thalamic nucleus |  |
| CPN | central pretectal nucleus |  |
| CPop | supraoptic commissure |  |
| Cpost | posterior commissure |  |
| Ctec | tectal commissure |  |
| Ctub | commissure of the posterior tuberculum |  |
| Cven | ventral rhombencephalic commissure |  |
| DAO | dorsal accessory optic nucleus |  |
| Dc | central zone of dorsal telencephalon area |  |
| DH | dorsal horn |  |
| DIL | diffuse nucleus of the inferior lobe |  |
| DiV | diencephalic ventricle |  |
| DIV | trochlear decussation |  |
| Dl | lateral zone of the dorsal telencephalon |  |
| Dm | medial zone of dorsal telencephalon |  |
| DON | descending octaval nucleus |  |
| DOT | dorsomedial optic tract |  |
| Dp | posterior zone of dorsal telencephalon area |  |
| DP | dorsal posterior thalamic nucleus |  |
| DR | dorsal root |  |
| DTN | dorsal tegmental nucleus |  |
| DV | descending trigeminal root |  |
| E | epiphysis |  |
| ECL | external cellular layer of olfactory bulb |  |
| EG | granular eminence |  |
| EmTl | lateral thalamic eminence |  |
| EmTm | medial thalamic eminence |  |
| EmTr | rostral thalamic eminence |  |
| ENd | entopeduncular nucleus, dorsal part |  |
| ENv | entopeduncular nucleus, ventral part |  |
| EW | Edinger-Westphal nucleus |  |
| Flv | ventral part of lateral funiculus |  |
| FR | habenulo-interpeduncular tract |  |
| Fv | ventral funiculus |  |
| GC | central gray |  |
| GL | glomerular layer of olfactory bulb |  |
| Had | dorsal habenular nucleus |  |
| Hav | ventral habenular nucleus |  |
| Hc | caudal zone of periventricular hypothalamus |  |
| Hd | dorsal zone of periventricular hypothalamus |  |
| Hv | ventral zone of periventricular hypothalamus |  |
| I (thalamus) | Intermediate thalamic nucleus |  |
| IAF | inner arcuate fibers |  |
| ICL | internal cellular layer of olfactory bulb |  |
| IMRF | intermediate reticular formation |  |
| IN | Intermediate nucleus |  |
| IO | inferior olive |  |
| IR | inferior raphe |  |
| IRF | inferior reticular formation |  |
| LC | locus coeruleus |  |
| LCa | caudal lobe of cerebellum |  |
| LFB | lateral forebrain bundle |  |
| LH | lateral hypothalamic nucleus |  |
| LLF | lateral longitudinal fascicle |  |
| LOT | lateral olfactory tract |  |
| LR | lateral recess of diencephalic ventricle |  |
| LRN | lateral reticular nucleus |  |
| MAC | Mauthner cell |  |
| MaON | magnocellular octaval nucleus |  |
| MFB | medial forebrain bundle |  |
| MFN | medial funicular nucleus |  |
| MLF | medial longitudinal fascicle |  |
| MON | medial octavolateralis nucleus |  |
| MOT | medial olfactory tract |  |
| NC | commissural nucleus of Cajal |  |
| NDV | nucleus of the descending trigeminal root |  |
| NI | isthmic nucleus |  |
| NIn | interpeduncular nucleus |  |
| NLL | nucleus of the lateral lemniscus |  |
| nLOT-a | nucleus of the lateral olfactory tract, anterior part |  |
| nLOT-i | nucleus of the lateral olfactory tract, intermediate part |  |
| nLOT-p | nucleus of the lateral olfactory tract, posterior part |  |
| NLV | nucleus lateralis valvulae |  |
| NMLF | nucleus of the medial longitudinal fascicle |  |
| NR | red nucleus |  |
| OENc | octavolateralis efferent neurons, caudal part |  |
| OENr | octavolateralis efferent neurons, rostral part |  |
| P | posterior thalamic nucleus |  |
| PC | posterior cerebellar tract |  |
| PCN | paracommissural nucleus |  |
| PGa | anterior preglomerular nucleus |  |
| PGc | caudal preglomerular nucleus |  |
| PGl | lateral preglomerular nucleus |  |
| PGm | medial preglomerular nucleus |  |
| PGZ | periventricular gray zone of optic tectum |  |
| PL | perilemniscal nucleus |  |
| PLLN | posterior lateral line nerve |  |
| PM | magnocellular preoptic nucleus |  |
| PMg | gigantocellular part of magnocellular preoptic nucleus |  |
| PO | posterior pretectal nucleus |  |
| POF | primary olfactory fiber layer |  |
| PON | posterior octaval nucleus |  |
| PPa | parvocellular preoptic nucleus, anterior part |  |
| PPd | periventricular pretectal nucleus, dorsal part |  |
| PPp | parvocellular preoptic nucleus, posterior part |  |
| PPv | periventricular pretectal nucleus, ventral part |  |
| PR | posterior recess of diencephalic ventricle |  |
| PSm | magnocellular superficial pretectal nucleus |  |
| PSp | parvocellular superficial pretectal nucleus |  |
| PTN | posterior tuberal nucleus |  |
| PVO | paraventricular organ |  |
| R | rostrolateral nucleus |  |
| RT | rostral tegmental nucleus |  |
| RV | rhombencephalic ventricle |  |
| SC | suprachiasmatic nucleus |  |
| SCO | subcommissural organ |  |
| SD | dorsal sac |  |
| SG | subglomerular nucleus |  |
| SGN | secondary gustatory nucleus |  |
| SGT | secondary gustatory tract |  |
| SO | secondary octaval population |  |
| SR | superior raphe |  |
| SRF | superior reticular formation |  |
| SRN | superior reticular nucleus |  |
| T | tangential nucleus |  |
| TBS | bulbo-spinal tract |  |
| TelV | telencephalic ventricles |  |
| TeO | optic tectum |  |
| TeV | tectal ventricle |  |
| TGN | tertiary gustatory nucleus |  |
| TL | longitudinal torus |  |
| TLa | lateral torus |  |
| TMCa | anterior mesencephalo-cerebellar tract |  |
| TMCp | posterior mesencephalo-cerebellar tract |  |
| TPM | pretecto-mammillary tract |  |
| TPp | periventricular nucleus of posterior tuberculum |  |
| TSc | central nucleus of semicircular torus |  |
| TSvl | ventrolateral nucleus of semicircular torus |  |
| TTB | tecto-bulbar tract |  |
| TTBc | crossed tecto-bulbar tract |  |
| TTBr | uncrossed tecto-bulbar tract |  |
| TVS | vestibulo-spinal tract |  |
| Val-g | lateral division of valvula cerebelli, granular layer |  |
| Val-m | lateral division of valvula cerebelli, molecular layer |  |
| Vam-g | medial division of valvula cerebelli, granular layer |  |
| Vam-m | medial division of valvula cerebelli, molecular layer |  |
| VAO | ventral accessory optic nucleus |  |
| Vas | vascular lacuna of area postrema |  |
| Vc | central nucleus of ventral telencephalon area |  |
| Vd-dd | dorsal zone of ventral telencephalon |  |
| Vd-vd | ventral zone of ventral telencephalon |  |
| Vdd | dorsal most zone of ventral telencephalon |  |
| Vl | lateral nucleus of ventral telencephalon area |  |
| VL | ventrolateral thalamic nucleus |  |
| VM | ventromedial thalamic nucleus |  |
| VOT | ventrolateral optic tract |  |
| Vp | postcommissural nucleus of ventral telencephalon area |  |
| Vs | supracommissural nucleus of ventral telencephalon area |  |
| Vv | ventral nucleus of ventral telencephalon area |  |
| ZL | zona limitans |  |
| III | oculomotor nerve |  |
| IIIm | oculomotor nucleus |  |
| IV | trochlear nerve |  |
| IVm | trochlear nucleus |  |
| V | trigeminal nerve |  |
| Vmd | trigeminal motor nucleus, dorsal part |  |
| Vmn | mesencephalic nucleus of the trigeminal nerve |  |
| Vmv | trigeminal motor nucleus, ventral part |  |
| Vmvr | ventral trigeminal motor root |  |
| Vsm | primary sensory trigeminal nucleus |  |
| Vsr | sensory root of the trigeminal nerve |  |
| VImc | caudal abducens nerve motor nucleus |  |
| VImr | rostral abducens nerve motor nucleus |  |
| VIILo | facial lobe |  |
| VIIm | facial motor nucleus |  |
| VIImr | facial motor root |  |
| VIIs | sensory root of the facial nerve |  |
| VIII | octaval nerve |  |
| IXLo | glossopharyngeal lobe |  |
| IXm | glossopharyngeal nerve motor nucleus |  |
| X | vagal nerve |  |
| XLo | vagal lobe |  |
| Xm | vagal motor nucleus |  |
| UnkD | unknown diencephalon |  |
| UnkMS | unknown mesencephalon |  |
| UnkR | unknown rhombencephalon |  |
| UnkSC | unknown spinal cord |  |
| UnkVT | unknown ventral telencephalon |  |
